# Utilization of Astaxanthin as a Synthetic Antioxidant Replacement for Emulsified Sausages

**DOI:** 10.3390/antiox10030407

**Published:** 2021-03-08

**Authors:** Jin-Kyu Seo, Rashida Parvin, Junyoung Park, Han-Sul Yang

**Affiliations:** 1 Division of Applied Life Science (BK21 Plus), Gyeongsang National University, 501 Jinju-daero, Jinju-si 52828, Korea; tjwlsrb7942@gmail.com (J.-K.S.); rakhiparvin@yahoo.com (R.P.); skyg5252@gmail.com (J.P.); 2 Institute of Agriculture and Life Science, Gyeongsang National University, 501 Jinju-daero, Jinju-si 52828, Korea

**Keywords:** emulsified sausage, astaxanthin, oxidation stability, color, texture properties

## Abstract

The aim of this study was to evaluate the effect of astaxanthin (AX) on the quality and sensory characteristics of emulsified pork sausages during cold storage. The changes of Peroxide value (PV), 2-thiobarbituric acid-reactive substances (TBARS), thiol content, texture profile analysis, instrumental color, and sensorial qualities were assessed on specific storage days. The emulsified sausages with added AX exhibited significantly (*p* < 0.05) higher redness values and total color differences (Δ*E*) on all storage days. Sensory values recorded the reddest color and greater overall acceptability scores to the sample with AX. In addition, AX had a significantly (*p* < 0.05) greater effect on PV, TBARS, and thiol content of sausages, compared with the control (CON). AX showed higher oxidation stability than CON for regression coefficient, and the level of inhibition of malondialdehyde formation was similar to that of butylated hydroxytoluene (BHT) on storage days. Synthetically, AX had a desirable consequence on antioxidant activity and color of emulsified sausages; therefore, it can be used as a multifunctional additive in emulsified pork sausages.

## 1. Introduction

Oxidation is one of the major causes of deterioration of food quality and is known to originate from fat, protein, and other oxidizing agents. They react with free radicals such as reactive oxygen species (ROS) and reactive nitrogen species (RNS) and cause acceleration of discoloration, formation of deleterious compounds, decrease in shelf life, and loss of nutrients, respectively. In addition, many studies have reported that the increasing level of oxidation leads to cell damage, causing critical diseases in the human body, such as modification of redox cell signaling and cardiovascular or chronic health issues [1,2].

Meat products are formulated with a large amount of fat, and consequently, oxidation of these products occur during production processes (trimming, grinding, and cooking). For that reason, manufacturers commonly use synthetic antioxidants, such as butylated hydroxytoluene (BHT), butylated hydroxyanisole (BHA), and tert-butylhydroquinone (TBHQ), to prevent oxidation. Although synthetic antioxidants are effective in the oxidation progress, they are potential source of carcinogens [3]. Ascorbic acid, however, can be used as a natural antioxidant in meat products and its effects have been reported. However, previous studies have shown that it acts as an antioxidant depending on the concentration, and the synergistic effect of its functional property is promoted when used in combination with another antioxidant [4]. Thus, in recent years, consumers have been demanding that natural antioxidants be used to reduce or replace synthetic antioxidants, and researchers have studied various possibilities for the application of natural sources. de Almeida et al. [5] suggested that jaboticaba peel extracts are efficient in preventing lipid oxidation and microbial activity and maintaining sensory characteristics in Bologna-type sausages. Fernandes et al. [6] reported that oregano extract added to cooked sausages resulted in lower lipid and protein oxidation, compared to sodium erythorbate. However, many studies have suggested the possibility of using antioxidants from various natural ingredients on meat products.

Astaxanthin (AX) is a group of xanthophylls in carotenoid, which is highly similar to the chemical structure of zeaxanthin, canthaxanthin, and β-carotene, and mainly obtained from salmon, crustacean, and algae; it is a natural red pigment. As is well-known from many studies, AX has various biological activities, such as antioxidant effects, anti-lipid peroxidation activity, and anticancer activity, among others [7,8]. The most important biological activity of AX is its antioxidant characteristic, which has been shown to be higher than α-tocopherol and other carotenoids [9]. AX is produced as a common dietary supplement and used in animal feeding. Recently, we found research related to AX applied to food, and Pogorzelska et al. [10] reported that *Haematoccocus pluvialis* extract rich in AX has significant lipid oxidation and DPPH scavenging activity in raw ground pork. The EFSA Panel on Nutrition [11] experimentally studied daily human intake of AX, and identified 8 mg AX per day from food supplements as applicable. It is logical that the application of AX as an antioxidant provides benefits in maintaining the oxidation stability of emulsified sausages. However, no studies have used AX as an antioxidant in emulsified sausages while comparing it with synthetic antioxidants. In this regard, this paper compared the effects of AX with commonly used antioxidants on oxidative stability, color, and texture properties of emulsified sausage at 21 days of storage.

## 2. Materials and Methods

### 2.1. Analytic Reagents Grade

All analytic reagents were purchased from Sigma Aldrich (Sigma-Aldrich, St. Louis, MO, USA)and the grades were ACS, HPLC, or GC.

### 2.2. Experimental Design and Manufacture of Emulsified Sausages

Fresh pork meat (mixture of loin and ham) and backfat, each from different animals, were purchased from a local market in Korea. Pork ham was trimmed to remove connective tissue and visible fat. Next, the meat and backfat were ground with a grinder (1787, LEM products, OH, USA) with plate (4 mm diameter), and the ground meat and backfat were mixed using a meat mixer (869, LEM products, West Chester Township, OH, USA). The mixture of meat and backfat was divided into four batches (10 kg for each batch) and stored at −80 °C after vacuum packaging. For this study, emulsified sausages were prepared using different formulas, and classified into three groups, designated as: negative control (without antioxidant), positive control (with BHT; 200 mg/kg), and treatment (with AX; 400 mg/kg). Each formulation was carried out four times.

AX was purchased as a powder type from Herblink Biotech Corporation, Shaanxi, China. Ground meat (557.1 g/kg), salt (14.6 g/kg), phosphate (4.6 g/kg), sugar (2.7 g/kg), monosodium glutamate (0.9 g/kg), and half of the iced water (68.5 g/kg) were placed in a bowl cutter (84145, Hobart Corporation, Troy, OH, USA) and mixed at 1725 rpm (knife) for 2 min. Then, starch (137.0 g/kg), backfat (146.1 g/kg), and the other half of the iced water (68.5 g/kg) were added and homogenized at the same speed for 5 min. The final temperature of the sausage batter was 8 °C. It was then stuffed into collagen casing (26 mm diameter) using a stuffer (838, LEM products, West Chester Township, OH, USA) and then clipped (HR-PS2, MAX^®^, Tokyo, Japan). Subsequently, the sausages were cooked in a convection steam oven (RCO-050E, KOSTEM, Gwangju, Korea) at 85 °C for 40 min. The internal temperature was 75 °C. After cooking, the sausages were chilled in an ice bath, packaged in oxygen permeable bags, and stored at 4 °C after being divided as per each experimental date (1, 7, 14, and 21 days).

### 2.3. Lipid Oxidation

PV was measured following a method by Shantha et al. [12]. Briefly, lipid was extracted by a chloroform and methanol mixture (2:1, *v*/*v*). 5 g of the sample was homogenized with 20 mL chloroform and methanol mixture, and filtered; the solvent was evaporated at room temperature. 30 mg of the extracted lipids were transferred into a glass test tube with 9.8 mL of chloroform and methanol mixture and vortexed for 5 s. In sequence, 30% ammonium thiocyanate and iron (II) solution (0.4 g barium chloride dehydrates, 0.5 g iron (II) sulfate, and 2 mL of 10 N HCl in 50 mL distilled water) were added to a glass test tube and vortexed for 5 s. The absorbance of the sample was read at 500 nm against a blank containing all reagents without lipids after left at room temperature for 5 min. The PV was expressed as meq of peroxide per kg of lipid.

TBARS of emulsified sausages was determined by a spectrophotometric method by Bozkurt et al. [13]. Two grams of minced sausages were taken and homogenized (T 25 D, IKA, Staufen, Germany) at 14,000 rpm for 30 s into a 50 mL conical tube with 27 mL of 4% perchloric acid. Homogenates were left at room temperature for 1 h and then filtrated with Whatman No. 1. 2 mL of the filtered solution with 2 mL of 20 mM TBA solution were pipetted into a glass test tube and kept at room temperature for 15 h after vortexing. The homogenate of distilled water was used as blank. The absorbance of the sample was measured against the blank at 531 nm. A standard curve was calculated using 1,1,3,3-tetraethoxypropane.

### 2.4. Protein Oxidation

The level of protein oxidation of the emulsified sausages was measured using Ellman’s reagent, DTNB [5,5-dithiobis (2-nitrobenzoic acid)] following a method employed by Berardo et al. [14] with slight modifications. Two grams of sausages was homogenized in 50 mL of 1% sodium dodecyl sulfate (SDS) in Tris buffer (pH 8.0), followed by incubation at 80 °C for 30 min in a water bath. The homogenate was then centrifuged at 7000× *g* for 20 min and filtered by Whatman No.1. 160 μL of 0.1 M Tris buffer (pH 8.0) and 40 μL of Ellman’s reagent (10 mM DTNB in Tris buffer) were added to an aliquot of supernatant (40 μL). A solution containing 40 μL of 1% SDS in Tris buffer, 40 μL of 10 mM DTNB, and 160 μL of Tris buffer was used as a reagent blank. The mixtures were left for 30 min in the dark with shaking at 250 rpm (MTS 2/4 D, IKA, Staufen, Germany), and absorbance was determined at 412 nm using a microplate reader (Epoch, Biotek Instruments, Inc., Winooski, VT, USA). The formula of Lambert–Beer (ε_412_ = 14,000 M^−1^ cm^−1^) was used to calculate thiol concentration, and the result was expressed in nmol thiol/mg protein. The bovine serum albumin standard curve was used to measure the protein concentration of the blank at 280 nm.

### 2.5. Texture Profile Analysis

Textural attributes were studied at room temperature with a texture analyzer (EZ-Test S-type, Shimadzu Corp., Kyoto, Japan). Before analysis, the sausages were left to equilibrate at room temperature for 30 min. Samples were taken from the central portion (2.5 cm × 2.5 cm) of each emulsified sausage. The conditions of texture analysis were as follows: pre-test speed 2.0 mm/s, test speed 2.0 mm/s, post-test speed 2.0 mm/s, maximum load 50 N, distance 6.0 mm, trigger force 5.0 g. The values for hardness (N) and cohesiveness were determined as described by Bourne et al. [15].

### 2.6. Instrumental Color Evaluation

Color measurements (CIE L*, a*, and b* value) were performed using a colorimeter (CR-300, Minolta, Osaka, Japan) with an 8-mm aperture size, light source D65, and angle of observation of 2°. The instrument was standardized with a white calibration plate (Y = 93.5, x = 0.3132, and y = 0.3198). Color measurements were performed at five points on the central parts of the surface of the emulsified sausage. Thickness was 20 mm, which prevents light from reflecting from the bottom. The total color difference (actual differences, Δ*E*), chroma, and hue angle were calculated according to Riazi et al. [16].

### 2.7. Sensory Evaluation

Sensory properties were evaluated by a panel of 20 trained members. Panelists evaluated the samples for color, flavor, chewiness, and overall acceptability using a 7-point hedonic scale, as described by Meilgaard et al. [17]. The emulsified sausages were individually provided to each panelist in sufficient quantities in a covered microwavable container. In addition, water to rinse the mouth was provided to each panelist to prevent cross-contamination among the samples. The test room ensured minimum distractions, noises, and odors. Each sample was randomly assigned a two-digit number so that the information of the sample was unknown. The total scores for each sample were calculated by adding the average score given by each panel member, for each of the five attributes.

### 2.8. Statistical Analysis

The data were collected for four batches at four different days per each treatment and storage day and presented as means with standard errors of means. Experimental analysis was performed in quadruplicates using the SAS program as version 9.4 (SAS Institute, Inc., Cary, NC, USA). The two-way ANOVA model (proc ANOVA) was performed with Duncan’s multiple range tests at a 95% significant level for comparison of treatment or storage day and their interaction. The linear regression model (proc reg) was conducted with an explanatory variable (storage day) and a response variable (quality trait) to determine the estimate of change over storage days.

## 3. Results and Discussion

### 3.1. Treatment Effect

Lipid oxidation was determined by hydroperoxide (primary product, PV) and malondialdehyde (secondary product, TBARS). It is also known that lipid oxidation is initiated from the labile hydrogen atom, and it produces oxides as per reaction stage. The primary stage of lipid oxidation produces hydroperoxide or results in loss of polyunsaturated fatty acid, and the secondary stage forms malondialdehyde or hydrocarbons. Therefore, lipid oxidation can be determined by measuring the several oxides in the oxidative stage [18,19]. The PV and TBARS of the emulsified sausages with or without specific antioxidants are shown in Table 1. Lipid oxidation by PV and TBARS had significant differences between treatments on some specific storage days (*p* < 0.05). In detail, the PV of AX did not see significant difference compared to the negative at 7 days of storage (*p* > 0.05), but was significantly lower than the negative control and AX on 14 and 21 days of storage (*p* < 0.05). Moreover, on day 14, the treatment group had insignificant differences in PV level with BHT (*p* > 0.05). It also showed the lowest TBARS during all storage days, similar to BHT (*p* < 0.05). A previous study mentioned that the limit of palatability of food containing fat is considered below 25 meq of active O_2_/kg [20]. The results after lipid oxidation suggest that AX had a positive effect on oxidative stability. 

The thiol content of the emulsified sausages with or without specific antioxidants is shown in Table 1. The range of values of thiol content in the emulsified sausages with or without antioxidants was indicated as 86.05 nmol/mg protein to 34.45 nmol/mg protein, which is comparable to the values found in Chinese-style sausages; only BHT had slightly high thiol content [21]. The reason for this is because the oxidation of lipid and protein is correlated with each other, as per previous reports [22], and BHT effectively inhibits lipid oxidation. Therefore, BHT had a higher thiol content than the other treatments. Thiol contents exhibited significant differences among treatments at all storage days (*p* < 0.05). The thiol content of BHT was significantly higher during all experiment periods than the other treatments (*p* < 0.05). On all experiment days, AX indicated insignificant differences in thiol content (*p* > 0.05), and were significantly higher in thiol content than CON (*p* < 0.05). According to Jiang et al. [23], many natural antioxidants have a positive effect on oxidative stability against lipid and protein oxidation, originating from their chemical structure and electron-donating ability. In addition, the beginning of lipid oxidation and protein is thought to be similar and mainly caused by hydroperoxide [22]. The oxidation of thiol groups is generated by forming disulfide bonds on the amino acid residue cysteine. Protein oxidation in meat products causes deterioration in quality, and oxidative damage of protein leads to textural degeneration.

The textural properties of emulsified sausages with/without antioxidants are presented in Table 2. The texture profile analysis of the emulsified sausages was determined by two parameters. Hardness (firmness) is one of the most important parameters of texture characteristics that evaluate the freshness of food, and cohesiveness is a significant property in meat-based products [24]. Hardness showed significant differences among treatments on all storage days (*p* < 0.05), and cohesiveness did not show significant differences over all storage days (*p* > 0.05). In hardness, the positive control was significantly higher than the other treatments on all storage days (*p* < 0.05). On all days, no significant difference in hardness value was shown between AX and the positive control (*p* > 0.05). Throughout the storage days, cohesiveness presented no significant difference in treatments (*p* > 0.05), but the cohesiveness of negative control was numerically higher than that of other treatments (*p* > 0.05). Our results are similar to that of Mercadante et al. [25], who observed that cooked sausages containing zeaxanthin or β-carotene were softer than control sausages with dextrose; the authors mentioned that the cooked sausages were more marked for decreasing hardness than the uncooked ones.

Color values of the emulsified sausages with/without antioxidants are presented in Table 2. On all storage days, color values such as lightness (L*), redness (a*), hue angle, and total color difference (Δ*E*) of the sausages had significant differences among treatments (*p* < 0.05), whereas yellowness (b*) and chroma were not seen to have significant differences among treatments (*p* > 0.05). AX displayed significantly (*p* < 0.05) lower L* and hue angle and higher a* and total color difference (Δ*E*) than the other treatments throughout the storage days. Color values such as L*, a*, and Δ*E* could be believed to the most instructive parameters for color changes [26]. The a* of AX was higher than that of the other treatments and thought to be produced from the origin of the color of astaxanthin. Bázan-Lugo et al. [27] reported that hue angle is an important criterion for determining color stability in meat products, and lower hue angle values indicate higher and more stable redness. However, our hue angle results showed that no change was observed during storage in all treatments, which means that all treatments retained their initial color. Therefore, the hue angle increased redness due to the addition of AX, so the AX showed a lower hue angle than the other control groups, as per the calculation formula. Color difference (Δ*E*), which was obtained from CIE L*, a*, and b* coordinates for all treatments, was different for actual visual color with/without antioxidants. In our results, the color difference value of AX was about 4–5 times higher than that of the positive control. According to Francis et al. [28], it can be seen that actual visual difference is when the color difference value of the measured color value is larger than 2. Therefore, the addition of 400 mg/kg astaxanthin to emulsified sausages would be able to increase the redness, and could be reason for greater consumer preference.

The result of sensory characteristics are shown in Table 3. The parameters of sensory characteristics are color, flavor, chewiness, and overall acceptability. Among the parameters, there were significant differences between the groups in color and overall acceptability, and AX showed the reddest color and highest overall acceptability (*p* < 0.05). Our results showed the same result as instrumental redness, and it could be considered that the difference was proved not only mechanically, but also visually. In addition, the highest overall acceptability of AX compared to the other groups was due to the difference in visual color, which had a positive effect on acceptability rather than the flavor and chewiness caused by AX.

### 3.2. Storage Effect

**Figure 1:**
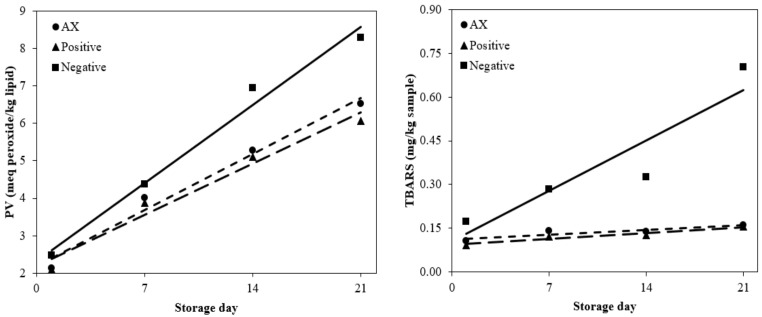
Effects of antioxidants on lipid oxidation of emulsified sausage. The solid lines are for the negative control, the large dashed line for the positive control, and the small dashed line for AX. Negative: without antioxidant; Positive: with 200 mg/kg mixture of BHT and BHA (1:1, *w*/*w*); AX, with 400 mg/kg astaxanthin.

Linear regression to express the level of inhibition for generation of PV and TBARS is shown in Table 4 and Figure 1. The slope values for the negative control, positive control, and AX from a linear regression coefficient of PV were 0.018, 0.005, and 0.002, respectively. The *R*^2^ of the negative control, positive control, and AX of PV were 0.84, 0.67, and 0.66, respectively. The slope value of AX was 1.6 times lower for PV than for the negative control. From the slope value, the addition of AX showed 1.6 times higher antioxidant activity than that of the negative control during cold storage.

The slope values for the negative control, positive control, and AX from the linear regression coefficient of TBARS were 0.020, 0.002, and 0.003, respectively. The *R*^2^ of the negative control, positive control, and AX of TBARS were 0.83, 0.45, and 0.47, respectively. Interestingly, the TBARS values of AX and positive control were not significantly difference during cold storage (*p* > 0.05). A previous study has shown that natural antioxidants show strong antioxidant activity in meat products, and their efficiency is more than that of synthetic antioxidants [29]. In particular, astaxanthin has a positive effect on oxidative stability against lipid oxidation in meat products, and the effectiveness of astaxanthin and other various natural antioxidants, such as natural material extracts [10,30], has been seen. Shah et al. [30] reported that AX showed a similar level of inhibition of lipid oxidation to BHT, and a similar level of inhibition. Currently, it is thought that this result was due to inhibition of hydroperoxide production, but further studies on the free radicals of BHT and AX should be carried out.

**Figure 2:**
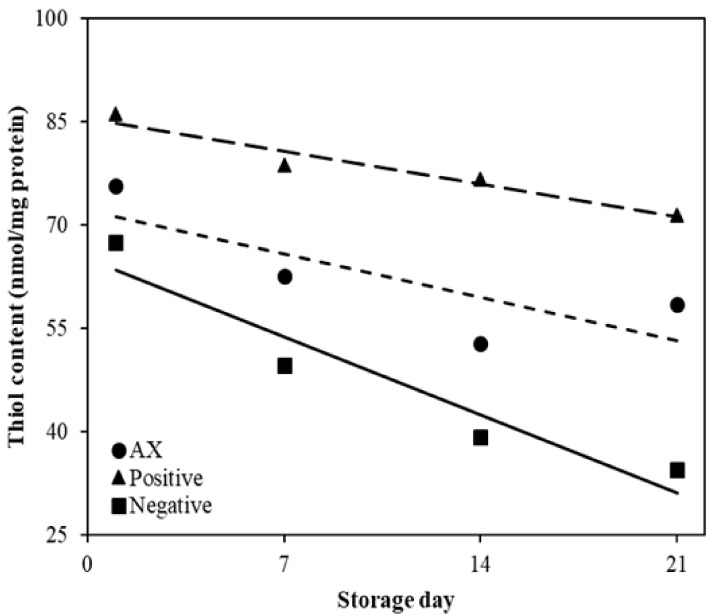
Effect of antioxidants on protein oxidation of emulsified sausages. The solid lines were for the negative control, the large dashed line for the positive control, and the small dashed line for AX. Negative: without antioxidant; Positive: with 200 mg/kg mixture of BHT and BHA (1:1, *w*/*w*); AX, with 400 mg/kg astaxanthin.

Linear regression was done to express the level of inhibition to loss of thiol (Table 4 and Figure 2). The slope values of the negative control, positive control, and AX from the linear regression coefficient of thiol content were −1.62, −0.90, and −0.68, respectively. The *R*^2^ of the negative control, positive control, and AX of thiol content were 0.73, 0.31, and 0.23, respectively. However, AX and the positive control were not observed to have significant differences in thiol content during storage periods (*p* > 0.05). Generally, the thiol group in protein is highly sensitive to oxidation in the existence of hydroperoxide, which is reduced by forming various compounds such as disulfide cross-link during the protein oxidation progress [22]. In addition, thiol content had significantly negative correlations with PV (correlation coefficient, −0.66; *p* < 0.001). Therefore, the reduction in thiol content could be affected by PV, and AX effectively inhibited the reduction of thiol content and the increase of PV during storage. Thus, AX was efficient in protein oxidation compared to the negative control.

**Figure 3:**
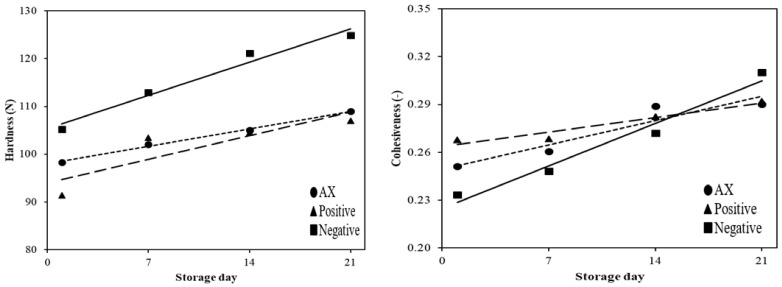
Effect of antioxidants on the textural properties of emulsified sausages. The solid lines are for the negative control, the large dashed lines for the positive control, and the small dashed lines for AX. Negative: without antioxidant; Positive: with 200 mg/kg mixture of BHT and BHA (1:1, *w*/*w*); AX, with 400 mg/kg astaxanthin.

Linear regression to express the changes for hardness and cohesiveness is presented in Table 4 and Figure 3. The slope values of CON, BHT, and AX from the linear regression coefficient of hardness were 0.99, 0.62, and 0.57, respectively. The *R*^2^ of the negative control, the positive control, and AX of hardness were 0.73, 0.71, and 0.72, respectively. The hardness values of all treatments were significantly different during cold storage (*p* < 0.05). However, cohesiveness values for all the treatments were not significantly different during storage periods (*p* > 0.05). With regard to hardness, the slope value of AX was lower than that of the negative control; especially, the slope value of AX was about 2.0 times lower than that of the negative control. Fernandes et al. [6] reported that the hardness of emulsified sausages and other cooked meat products increased during cold storage. This attribute could be due to the oxidative damage of proteins, which leads to the aggregation and complex formation of protein by cross-links as well as an influence on protein solubility [6,31]. In addition, Ganhão et al. [32] reported that the increase of hardness in emulsified burger patties was due to protein oxidation during cold storage. Therefore, the results suggest that the use of AX effectively inhibited the increase of hardness during cold storage.

The linear regression result about color are not presented as there were no significant difference in storage effects. The fact that the regression results had no significant difference means that the color of the sausages remained stable during the storage period. The redness values for the negative and positive controls were indicated by −1.45 to −1.95 during the storage period; however, AX had values of 3.04 to 3.25 during the storage period. According to Seo et al. [33], 0.1% *Caesalpinia sappan* L. extract-treated cooked pork sausages showed that the redness values ranged from 3.02 to 3.75 at 15 days during cold storage, which is similar to our results. Nonetheless, the author of [6] reported that sheep sausages with oregano extract had discolored, especially defined by loss of redness from 15.54 to 7.14 at room temperature during storage. This could be explained by different conditions such as the ratio of air, moisture permeability of the packing bag, and storage temperature. In fact, the authors used an oxygen-water permeable bag and storage temperature at 20 ± 2 °C in dark conditions.

As aforementioned, astaxanthin is a carotenoid and fat soluble. Thus, it can be dissolved in pork fat and can act as a colorant in emulsified sausages. Additionally, carotenoids are dyes in oil or fats but pigments in water. In other words, a dye or pigment is determined by its solubility or not in a given medium [34]. Therefore, astaxanthin would be more suitable for dye-based rather than pigment-based action in emulsified sausages.

## 4. Conclusions

This study was carried out to investigate the effect of AX on the qualitative characteristics of emulsified pork sausages during cold storage. The addition of AX at 400 mg/kg to emulsified sausages had positive effects on oxidative stability against lipid and protein oxidation and textural properties, and these effects were similar to the addition of BHT at 200 mg/kg. Furthermore, improved appearance quality, like redness and visual color during the treatments, might be due to the addition of AX. In summary, AX as a functional ingredient could play an important role in improving the physical and sensory qualities of emulsified sausages by having positive effects on color, textural properties, and oxidation stability. Therefore, it could be used as a natural multi-functional ingredient for antioxidant efficiency and color development in emulsified pork sausages.

## Figures and Tables

**Table 1:** Lipid and protein oxidation evaluation during cold storage of emulsified sausages with different antioxidants.

Traits	Treatments ^1^	Storage Days			
		1	7	14	21
Peroxide value (PV, meq peroxide/kg lipid)	Negative control	2.47 ^A^	4.37 ^A^	6.94 ^A^	8.29 ^A^
	Positive control	2.09 ^C^	3.87 ^B^	5.11 ^C^	6.07 ^D^
	AX	2.14 ^AB^	4.01 ^AB^	5.27 ^C^	6.52 ^C^
	SEM	0.02	0.26	0.18	0.33
TBARS (mg MDA/kg sample)	Negative control	0.17 ^A^	0.28 ^A^	0.33 ^B^	0.70 ^A^
	Positive control	0.09 ^B^	0.12 ^C^	0.13 ^C^	0.16 ^B^
	AX	0.11 ^B^	0.14 ^C^	0.14 ^C^	0.16 ^B^
	SEM	0.01	0.01	0.01	0.02
Thiol content (nmol/mg protein)	Negative control	67.53 ^C^	49.61 ^C^	39.21 ^C^	34.45 ^C^
	Positive control	86.05 ^A^	78.69 ^A^	76.64 ^A^	71.40 ^A^
	AX	75.76 ^B^	62.68 ^B^	58.53 ^B^	52.78 ^B^
	SEM	1.09	1.81	1.18	1.12

^A–D^ Means within a column with different superscripts are significantly different (*p* < 0.05). ^1^ Negative: without antioxidant; Positive: with 200 mg/kg mixture of BHT and BHA (1:1, *w*/*w*); AX, with 400 mg/kg astaxanthin.

**Table 2:** Textural properties and instrumental color evaluation during cold storage of emulsified sausages with different antioxidants.

Traits	Treatments	Storage Days			
		1	7	14	21
Hardness (N)	Negative control	105.25 ^A^	112.88 ^A^	120.52 ^A^	124.89 ^A^
	Positive control	91.33 ^B^	103.48 ^B^	104.49 ^B^	107.44 ^C^
	AX	100.73 ^AB^	102.55 ^B^	106.31 ^B^	109.90 ^BC^
	SEM	1.84	1.55	2.31	1.16
Cohesiveness	Negative control	0.31	0.29	0.29	0.31
	Positive control	0.27	0.29	0.28	0.27
	AX	0.25	0.27	0.28	0.24
	SEM	0.03	0.03	0.02	0.02
L*	Negative control	77.91 ^A^	78.18 ^A^	77.90 ^A^	78.35 ^A^
	Positive control	78.90 ^A^	79.10 ^A^	78.83 ^A^	79.49 ^A^
	AX	73.69 ^B^	74.31 ^B^	74.38 ^B^	74.20 ^B^
	SEM	0.41	0.73	0.54	0.56
a*	Negative control	−1.68 ^B^	−1.73 ^B^	−1.65 ^B^	−1.95 ^B^
	Positive control	−1.78 ^B^	−1.65 ^B^	−1.45 ^B^	−1.76 ^B^
	AX	3.25 ^A^	3.20 ^A^	3.20 ^A^	3.04 ^A^
	SEM	0.20	0.22	0.42	0.19
b*	Negative control	15.75	15.99	15.88	16.18
	Positive control	15.91	15.99	15.78	16.06
	AX	15.77	15.92	16.16	16.07
	SEM	0.40	0.34	0.31	0.55
Chroma	Negative control	15.84	16.09	15.96	16.30
	Positive control	16.01	16.08	15.85	16.15
	AX	16.10	16.24	16.47	16.35
	SEM	0.40	0.34	0.30	0.36
Hue angle	Negative control	95.99 ^A^	96.08 ^A^	95.88 ^A^	96.82 ^A^
	Positive control	96.29 ^A^	95.78 ^A^	95.15 ^A^	96.13 ^A^
	AX	78.39 ^B^	78.67 ^B^	78.88 ^B^	79.36 ^B^
	SEM	0.69	0.74	0.69	0.61
Total color difference (Δ*E*)	Negative control	0.00 ^C^	0.00 ^B^	0.00 ^C^	0.00 ^C^
	Positive control	1.23 ^B^	1.26 ^B^	1.08 ^BC^	1.37 ^B^
	AX	6.55 ^A^	6.34 ^A^	6.03 ^A^	6.54 ^A^
	SEM	0.30	0.43	0.42	0.34

^A–C^ Means within a column with different superscripts are significantly different (*p* < 0.05). Negative: without antioxidant; Positive: with 200 mg/kg mixture of BHT and BHA (1:1, *w*/*w*); AX, with 400 mg/kg astaxanthin.

**Table 3:** Sensory properties of emulsified sausages with different antioxidants.

Groups ^1^	Parameters ^2^			
	Color	Flavor	Chewiness	Overall Acceptability
Negative control	3.28 ^b^	5.40	6.20	5.16 ^d^
Positive control	3.28 ^b^	5.32	6.72	5.84 ^c^
AX	4.40 ^a^	5.64	6.16	6.76 ^a^
SEM	0.12	0.15	0.34	0.21

^a–d^ Means within a column with different superscripts are significantly different (*p* < 0.05). ^1^ Negative: without antioxidant; Positive: with 200 mg/kg mixture of BHT and BHA (1:1, *w*/*w*); AX, with 400 mg/kg astaxanthin. ^2^ Based on a 7-point intensity scale (1 = dislike extremely or extremely light/bland/dry; and 7 = like extremely or extremely reddish/intense/juice).

**Table 4:** Linear regression of lipid and protein oxidation and textural properties of emulsified sausages with different antioxidants during cold storage.

Dependent Variable	Negative Control			Positive Control			AX		
	*R* ^2^	Coefficient	*p*-Value	*R* ^2^	Coefficient	*p*-Value	*R* ^2^	Coefficient	*p*-Value
Lipid oxidation									
Peroxide value (PV, meq peroxide/kg lipid)	0.84	0.018	***	0.67	0.005	**	0.66	0.002	**
TBARS (mg MDA/kg sample)	0.83	0.020	***	0.82	0.002	***	0.76	0.003	***
Protein oxidation									
Thiol content (nmol/mg protein)	0.90	−1.62	***	0.80	−0.68	***	0.84	−1.08	***
Texture profile analysis									
Hardness (N)	0.78	0.99	***	0.72	0.62	***	0.81	0.57	***
Cohesiveness	NS								

NS, no significance; **, significance level at <0.01; ***, significance level at <0.001. Negative: without antioxidant; Positive: with 200 mg/kg mixture of BHT and BHA (1:1, *w*/*w*); AX, with 400 mg/kg astaxanthin.

## Data Availability

Not applicable.
